# Meet Caregivers Where They Are: A Remote Intervention Connecting Caregivers to Community Resources

**DOI:** 10.1093/geroni/igab046.2393

**Published:** 2021-12-17

**Authors:** Katherine Thompson, Victoria Winslow, El Pinkerton, Elbert Huang, Maria de Ornelas, Soo Borson, Jason Molony, Stacy Lindau

**Affiliations:** 1 University of Chicago, Chicago, Illinois, United States; 2 The University of Chicago, Chicago, Illinois, United States; 3 University of Washington School of Medicine, Seattle, Washington, United States; 4 The University of Chicago Medicine, Chicago, Illinois, United States

## Abstract

Informal caregivers of people with Alzheimer’s disease and related dementias (ADRD) are a vulnerable, often isolated population with high rates of financial strain and need for community resource supports. Little is known about how best to connect these caregivers to resources, especially during the COVID-19 pandemic. CommunityRx-Caregiver is an evidence-based intervention that connects caregivers to community resources for basic needs, wellness, and caregiving. Using preliminary data from a randomized trial of CommunityRx-Caregiver (N=344), we examined caregivers’ baseline confidence in finding community resources and their engagement in the CommunityRx-Caregiver intervention. Caregivers enrolled December 2020-February 2021 (n=26) received (1) personalized lists of community resources via text message (HealtheRx), (2) access to an online resource portal (FindRx) and (3) automated texts offering support for finding resources. Most caregivers were female (65%), Black (92%), >60 years old (64%) and 44% reported very good or excellent health. Nearly half of caregivers (46%) were completely confident in finding community resources. Overall, 81% of caregivers engaged with a text message or the FindRx. Nearly two-thirds (65%) of caregivers responded to at least one text message. More than a quarter (27%) used the FindRx tool; 5/7 of those shared FindRx resources with others. Caregivers sought resources including in-home personal care, exercise classes and support groups. Caregivers of people with ADRD, many of whom had low confidence in finding resources, engaged with a multi-modal information technology-based intervention to obtain community resource support. These preliminary findings suggest caregivers were receptive to a remotely-delivered community referral intervention during the COVID-19 pandemic.

